# Reviewers in 2024

**DOI:** 10.1098/rsos.250498

**Published:** 2025-05-07

**Authors:** Wendy Hall

**Affiliations:** ^1^ Editor-in-Chief

The tradition established by the Royal Society Publishing of publishing an annual list of thanks to peer reviewers who served in the previous calendar year continues in the below article.

The Editors and journal administrative team of *Royal Society Open Science* would like to thank all reviewers who contributed their time by refereeing manuscripts submitted last year. We remain extremely grateful for your support and expertise and hope you will serve in 2025 and beyond.

Reviewers value recognition of their efforts by the journal and through services such as the Web of Science Reviewer Recognition Service, which is integrated with *Royal Society Open Science*.

To recognize and support our peer reviewers, we list the names of all reviewers who served in 2024 and have opted to be included.

This article is made permanent and citable by its digital object identifier (DOI). We encourage you to quote your contribution in your applications for tenure and grants or other forms of research assessment. Where you have provided it, we have included your ORCID, so your contribution can be unambiguously assigned to you.

On behalf of all the Editors and editorial office, thanks once again to all of our reviewers past, present and future, and we look forward to your continued support of our journal.

Aakash M

Abdel Hameed R

https://orcid.org/0000-0003-2785-894X

Aboelalla M

https://orcid.org/0000-0002-8579-0093

Abourachid A

https://orcid.org/0000-0002-7238-2795

Acharya A

https://orcid.org/0000-0002-6184-3357

Adkins-Regan E

Aedo C

Aeles J

https://orcid.org/0000-0003-1514-4958

Aggleton J

Agostini S

https://orcid.org/0000-0001-9040-9296

Agrawal A

https://orcid.org/0000-0003-0095-1220

Ahn YY

Aich U

https://orcid.org/0000-0003-2576-0922

Ainley D

Ajates R

Akay T

Al-Kubaisi MHD

Al-Mahamad L

https://orcid.org/0000-0002-2783-8669

Alam K

https://orcid.org/0000-0002-3528-0044

Alam M

https://orcid.org/0000-0002-5188-1130

Alam MDS

Alarcon DS

Albarella U

Alexander A

Ali A

https://orcid.org/0000-0002-2914-0934

Ali M

Aliotta C

Alksne M

Allan B

https://orcid.org/0000-0002-5991-9711

Allen C

Altintig E

Altshuler D

https://orcid.org/0000-0002-1364-3617

Amat M

Amici F

Ammarullah MI

https://orcid.org/0000-0002-8845-7202

Amos M

https://orcid.org/0000-0003-1680-5535

Amuzescu B

Anderson A

https://orcid.org/0000-0002-2536-4383

Andrello M

https://orcid.org/0000-0001-7590-2736

Andrews T

Anghel G

https://orcid.org/0000-0001-5054-2041

Antcliffe J

https://orcid.org/0000-0001-8639-6998

Antle M

Anđelković M

Aoki K

https://orcid.org/0000-0002-5986-9849

Apeji YE

Araújo Marreiro Varela S

https://orcid.org/0000-0002-9947-4024

Arellano-Veliz N

Arias M

Arias MJL

Arima H

Arntzen JW

https://orcid.org/0000-0003-3229-5993

Arroyo-Machado W

https://orcid.org/0000-0001-9437-8757

Arshad M

Asally M

https://orcid.org/0000-0002-8273-7617

Asami T

Asghar Z

Asif MB

https://orcid.org/0000-0003-0126-4617

Astolfi ML

Atance D

Atari M

Atkinson M

Atzil S

Avila C

Azarian T

https://orcid.org/0000-0002-8611-5241

Aze T

Azevedo F

Azhagapillai P

Azizi E

Babbar B

Badawi A

Badihi G

Bahadori-Jahromi A

Bahrami M

Bai F

Bai M

Bai S

Bailey E

Bailey J

Bain A

https://orcid.org/0000-0002-6111-7013

Bain J

Bak-Coleman J

https://orcid.org/0000-0002-7590-3824

Balasis G

https://orcid.org/0000-0001-7342-0557

Balcarcel A

https://orcid.org/0000-0002-3826-834X

Baliga V

https://orcid.org/0000-0002-9367-8974

Ballen-Moreno F

Balluffi-Fry J

https://orcid.org/0000-0002-2365-1055

Bamford J

https://orcid.org/0000-0002-8204-7915

Bandyopadhyay D

Banerjee B

Bansal S

https://orcid.org/0000-0002-1740-5421

Bao S

https://orcid.org/0000-0003-1295-3389

Bara J

Baragli P

Barakos GN

Barbier E

https://orcid.org/0000-0002-5354-3995

Bard K

Bari V

https://orcid.org/0000-0002-5346-2677

Barnby J

https://orcid.org/0000-0001-6002-1362

Baron A

Barrett P

Bartos L

https://orcid.org/0000-0003-2667-5930

Basanovic J

Basanta D

https://orcid.org/0000-0002-8527-0776

Bassett D

https://orcid.org/0000-0002-6183-4493

Bauer J

https://orcid.org/0000-0003-3635-1235

Bavolar J

https://orcid.org/0000-0003-0179-7261

Bazhydai M

Beatty B

https://orcid.org/0000-0002-5464-0041

Beatty MW

Beck R

https://orcid.org/0000-0002-7050-7072

Becker D

https://orcid.org/0000-0003-4315-8628

Beehner J

Beetz MJ

https://orcid.org/0000-0001-6568-8596

Beiming T

Beja-Pereira A

https://orcid.org/0000-0002-1607-7382

Bekvalac J

Belk M

Bellardini F

Bellows S

https://orcid.org/0009-0002-5549-1292

Belmaker M

Belmonte Beitia J

https://orcid.org/0000-0002-5003-1150

Benard A

Bennett MT

Bennewitz R

Berardo A

https://orcid.org/0000-0002-2346-0507

Berg M

https://orcid.org/0000-0003-1403-1978

Bergelson E

Bergman T

https://orcid.org/0000-0002-9615-5001

Bertelloni F

Berthaume M

https://orcid.org/0000-0003-1298-242X

Bertolucci C

Berton P

Bertram J

Bester-Rogac M

Bestwick J

Bett B

Bevan E

Bevan S

Bhaduri S

Bhattacharjee D

https://orcid.org/0000-0002-4555-7983

Bhowmick S

Bicknell R

https://orcid.org/0000-0001-8541-9035

Bidula S

Bielby J

https://orcid.org/0000-0003-4586-0429

Biggs P

Bikas R

Billinghurst M

Bimpisidis Z

https://orcid.org/0000-0002-8819-7957

Birhane A

Birkbeck M

Birn-Jeffery A

Biuw M

https://orcid.org/0000-0001-8051-3399

Blawas A

Blonder B

Blount JD

Blyuss K

Blázquez J

Boero M

https://orcid.org/0000-0002-5052-2849

Bolsen T

Bond C

Bonzi L

https://orcid.org/0000-0003-4320-4348

Borge B

Borghi J

https://orcid.org/0000-0001-9570-4163

Borowski Z

https://orcid.org/0000-0003-4109-5205

Borzi A

Boulebd H

https://orcid.org/0000-0002-7727-8583

Boulinier T

Bouquet C

https://orcid.org/0000-0003-4228-3291

Bouyeure A

Bowers D

Bowles A

Boyce M

Boyce V

https://orcid.org/0000-0002-8890-2775

Boyers M

https://orcid.org/0000-0001-7129-8949

Boyle A

Bramble D

Brand J

https://orcid.org/0000-0003-3312-941X

Brandl H

Braun C

Braun H

Bravo G

https://orcid.org/0000-0003-2837-0137

Brayne C

Brazeau M

https://orcid.org/0000-0002-0650-1282

Brazil K

Brecht M

Breedveld M

Bremhorst A

https://orcid.org/0000-0003-2185-3101

Bretman A

https://orcid.org/0000-0002-4421-3337

Breznau N

https://orcid.org/0000-0003-4983-3137

Brinkman D

Britt N

Brookfield J

https://orcid.org/0000-0002-0509-0274

Brown C

https://orcid.org/0000-0002-0210-1820

Browne L

Bruckner R

Brysbaert M

Brønnvik H

https://orcid.org/0000-0002-3061-0630

Buckeridge J

Buckner J

Buffa V

Bufo MR

Bufon C

Bugnyar T

Bunney E

https://orcid.org/0000-0002-7905-2975

Burkett J

https://orcid.org/0000-0002-6357-5499

Bury T

https://orcid.org/0000-0003-1595-9444

Bærum K

Çağlayan R

Caldwell M

https://orcid.org/0000-0002-2377-3925

Calude C

https://orcid.org/0000-0002-8711-6799

Camacho EAR

Camargo C

Campillo-Funollet E

Camps I

Canoville A

https://orcid.org/0000-0002-9798-176X

Cappa F

https://orcid.org/0000-0002-3510-8885

Capraro V

https://orcid.org/0000-0002-0579-0166

Capriati V

Carbonara P

Carcaud J

https://orcid.org/0000-0003-3672-9963

Cardeñosa D

Cardini AL

Carere C

https://orcid.org/0000-0003-1644-2113

Cargnelutti F

Carlson N

https://orcid.org/0000-0002-7785-1247

Carragee E

Carter C

Carvajalino JM

https://orcid.org/0000-0002-2404-0037

Cass J

Cassidy T

Castaldelli E

https://orcid.org/0000-0003-2946-8697

Catmur C

Cauchard L

Cayado P

Cea P

Ceci SJ

Cecilia JA

Celiker E

https://orcid.org/0000-0003-4988-7901

Cenci S

https://orcid.org/0000-0001-6843-468X

Ceron T

Chaikin S

Chaine A

Chaiyarat R

Chakraborty S

Chala D

Chalkias D

https://orcid.org/0000-0003-3480-0666

Chang P

Chao W

Chapman J

https://orcid.org/0000-0002-7475-4441

Charafeddine R

Charalabidis A

Chatterjee S

Chattopadhyaya S

Chaudhary S

Chegel R

Chen C

Chen J

Chen K-N

Chen W-B

Chen WB

Chen Z

Cheng B

Chermette H

Chernyavsky I

https://orcid.org/0000-0003-0284-9318

Cheung HN

Chipman A

Chirichella R

Chow J

Christodoulides C

Christoforides E

Chumachenko D

https://orcid.org/0000-0003-2623-3294

Chutia A

https://orcid.org/0000-0002-5897-1729

Ciatto G

Cieri R

https://orcid.org/0000-0001-6905-9148

Cirelli LK

Clark D

Clayton J

Clemente C

https://orcid.org/0000-0001-8174-3890

Clements C

https://orcid.org/0000-0002-0637-1625

Clunes M

https://orcid.org/0000-0003-4565-3235

Cobb S

https://orcid.org/0000-0002-8360-8024

Coco MI

Codorniu L

Cohen K

Colasante T

Cole NL

https://orcid.org/0000-0002-6034-533X

Collier J

Collins BA

Collins S

https://orcid.org/0000-0003-0059-6900

Colman A

Colman E

Colombi A

https://orcid.org/0000-0001-6282-3326

Colombo M

https://orcid.org/0000-0002-2574-4888

Combariza MY

Cong P

Conway P

Coogan A

Coon R

Cooper J

Copilas-Ciocianu D

Corda C

Cordiner J

Corlett P

https://orcid.org/0000-0002-5368-1992

Cornil C

https://orcid.org/0000-0002-5536-7753

Cortell-Nicolau A

Cortellini V

Cossin-Sevrin N

Costa-Font J

Cota W

Cotter S

https://orcid.org/0000-0002-3801-8316

Cousineau D

Cox C

Creel S

Croitor R

Crook B

Crosato E

https://orcid.org/0000-0002-7629-774X

Crowe-Riddell J

https://orcid.org/0000-0003-2794-2914

Cruz RMS

Csata E

Curnick D

https://orcid.org/0000-0002-3093-1282

Curth S

Curtin L

https://orcid.org/0000-0002-4083-3803

Cury J

Cusano D

D'Agostino A

da Silva L

Dabaghi M

Daehlin T

Dahl S

Dal Pesco F

https://orcid.org/0000-0003-2326-1185

Dalmaijer E

https://orcid.org/0000-0003-3241-0760

Dalwadi M

https://orcid.org/0000-0001-5017-2116

Damer Bruce

Damineli D

https://orcid.org/0000-0002-9911-2891

Daniels K

https://orcid.org/0000-0001-8134-6764

Das P

Datta S

https://orcid.org/0000-0003-2198-7900

Daud KM

Dautriche I

https://orcid.org/0000-0002-2297-985X

Daversa D

Davies J

https://orcid.org/0000-0001-7635-4815

Davies K

https://orcid.org/0000-0001-6060-2394

Davis DS

Davis J

Dawson SJ

de Baaij JHF

de Carvalho ACPLF

de Graaf B

De Houwer J

https://orcid.org/0000-0003-0488-5224

de Kock W

de la Vega A

De Nicola G

De Silva R

https://orcid.org/0000-0003-0955-6366

De Sojo S

de Zwaan D

Deak A

Dean C

https://orcid.org/0000-0003-4287-4390

Deffner D

https://orcid.org/0000-0002-1649-3861

Del Missier F

Dellisanti W

Delsett L

https://orcid.org/0000-0002-6806-1411

Des Marteaux L

https://orcid.org/0000-0002-0461-2704

Deshpande S

Deutschmann IM

https://orcid.org/0000-0002-3512-261X

Diagboya PN

Diaz M

https://orcid.org/0000-0002-6384-6674

Dick T

https://orcid.org/0000-0002-7662-9716

Dickenson NE

Didziokas M

DiLeo M

https://orcid.org/0000-0003-0101-5274

Dimdiņš Ģ

Dinets V

https://orcid.org/0000-0002-7423-7487

Ding S

Diqi M

Dissegna A

https://orcid.org/0000-0002-4333-3353

Dixon D

Dixon S

https://orcid.org/0000-0002-6098-481X

Diykh M

Dobrovolny H

https://orcid.org/0000-0003-3592-6770

Dogan IS

Doherty J-F

https://orcid.org/0000-0003-4766-9417

Domic A

Donelson J

Dong TG

Doolittle E

Dopheide A

Dorin J

Doube M

https://orcid.org/0000-0002-8021-8127

Dougherty L

https://orcid.org/0000-0003-1406-0680

Drake J

https://orcid.org/0000-0002-9851-379X

Dreber A

https://orcid.org/0000-0003-3989-9941

Du A

https://orcid.org/0000-0002-7381-5089

Duarte S

Duckett D

Ductor L

Duffield K

Duncan G

Dunlop J

https://orcid.org/0000-0002-0179-6640

Dunn J

https://orcid.org/0000-0002-1189-1908

Dylewsky D

https://orcid.org/0000-0003-4904-1391

Dzewaltowski A

https://orcid.org/0000-0003-1999-0246

d’Ingeo S

https://orcid.org/0000-0002-6164-2598

Eales O

https://orcid.org/0000-0002-8086-4495

Early C

https://orcid.org/0000-0003-0308-2710

Ebert M

Eby W

Eddy C

https://orcid.org/0000-0001-7821-1121

Efimova SS

Egide K

Ehrich D

https://orcid.org/0000-0002-3028-9488

Eigenbrod F

Eklund A

El Hammoudani Y

El-Nemr A

El-Shaheny R

Elgesem D

Eliasy A

https://orcid.org/0000-0002-4473-1900

Elkassimi M

Elliott M

Ellison J

Elsayed O

https://orcid.org/0000-0002-7203-4846

Emam H

Emery Thompson M

https://orcid.org/0000-0003-2451-6397

Emmert-Streib F

Enderling H

Eriksson A

Esattore B

https://orcid.org/0000-0003-1024-794X

Escalera D

Espin Noboa LE

https://orcid.org/0000-0002-3945-2966

Esteve J

https://orcid.org/0000-0003-2806-2695

Estrada-Saldivar NA

Ettahiri Y

Evans S

Evers S

Evin A

https://orcid.org/0000-0003-4515-1649

Eşkazan AE

https://orcid.org/0000-0001-9568-0894

Fadda C

Fairbanks E

Faizan Nazar M

Fakoor M

Falcon-Cortes A

Falcó C

https://orcid.org/0000-0001-9832-2697

Falkenberg M

Farine D

Farrahi K

Farrar V

Farrell T

Fattahi M

Fell D

Feng T

Fenton A

https://orcid.org/0000-0002-7676-917X

Fernandes D

Fernandez-Duque E

Fernandez-Garcia J

Fernández Jalvo Y

Ferreira R

https://orcid.org/0000-0001-7774-5252

Fialko K

https://orcid.org/0000-0002-6254-2951

Fichtel C

https://orcid.org/0000-0002-8346-2168

Fiedler G

Figueiredo A

Figueirido B

https://orcid.org/0000-0003-2542-3977

Fine J

Fiorito G

https://orcid.org/0000-0003-2926-9479

Fischer J

https://orcid.org/0000-0002-5807-0074

Fischer V

https://orcid.org/0000-0002-8808-6747

Fitneva S

Fjelldal M

https://orcid.org/0000-0001-6642-906X

Flanagan S

https://orcid.org/0000-0002-2226-4213

Flores K

https://orcid.org/0000-0002-4000-6971

Fonkou M

Fonseca A

Foote AD

Forgoston E

https://orcid.org/0000-0003-0694-9981

Fortunato L

https://orcid.org/0000-0001-8546-9497

Fragaszy D

https://orcid.org/0000-0002-4010-4993

Francesconi M

Frank M

https://orcid.org/0000-0001-9487-9359

Franzem T

Frasca P

Fraser D

https://orcid.org/0000-0002-0228-2617

Frasier T

https://orcid.org/0000-0002-3055-0199

Friedman R

Friston K

https://orcid.org/0000-0001-7984-8909

Frommen J

https://orcid.org/0000-0002-1752-6944

Fröhlich M

https://orcid.org/0000-0002-1948-7002

Fu Y

https://orcid.org/0009-0002-1582-2771

Fuglevand A

https://orcid.org/0000-0002-4349-6478

Fukada Y

https://orcid.org/0000-0003-1058-5877

Fukushima M

https://orcid.org/0000-0002-8809-7892

Furuichi T

Furukawa S

https://orcid.org/0000-0002-5833-7891

G-Guzmán E

https://orcid.org/0000-0003-2781-287X

G.P. RS

https://orcid.org/0000-0002-3634-2171

Gabor C

https://orcid.org/0000-0001-7584-1451

Gado W

Gagniuc Paul

Gallagher J

https://orcid.org/0000-0001-5705-1325

Gallagher K

Galteri G

Gamble A

Gan Q

Gandolfo M

https://orcid.org/0000-0003-1505-3191

Ganguly S

Garssen B

Garwood R

https://orcid.org/0000-0002-2803-9471

Gasparini C

https://orcid.org/0000-0001-9172-1142

Gates TA

Gautam H

https://orcid.org/0000-0001-8289-5562

Gaynor K

https://orcid.org/0000-0002-5747-0543

Gebeyehu BT

https://orcid.org/0000-0001-7890-9466

Gebhardt-Henrich S

Gedara P

Genwa K

Georgalis G

Gerisch A

Gerlee P

Geronikaki A

Gesmalla A

Ghamari kargar P

https://orcid.org/0000-0002-6284-2874

Gianechini F

Gianturco F

Gibbon D

Gibbons M

https://orcid.org/0009-0000-3221-6031

Gibson B

https://orcid.org/0000-0001-9620-0063

Gifford M

https://orcid.org/0000-0003-1263-0010

Gill T

https://orcid.org/0000-0001-9839-4113

Gillis R

https://orcid.org/0000-0002-2370-7311

Gilman R

https://orcid.org/0000-0003-2000-7966

Girard-Buttoz C

https://orcid.org/0000-0003-1742-4400

Girgis A

https://orcid.org/0000-0003-4407-9745

Giribabu L

Girirajan S

Gizzi A

https://orcid.org/0000-0001-5350-8156

Gjini E

https://orcid.org/0000-0002-0493-3102

Glemarec G

https://orcid.org/0000-0003-1801-3179

Glikman JA

Goblirsch M

Godoy I

Goenka A

Goh K-L

Golightly A

Gomes S

https://orcid.org/0000-0002-8731-367X

Gomez-Mestre I

https://orcid.org/0000-0003-0094-8195

Gomha SM

Goncalves B

Gonnerman M

https://orcid.org/0000-0002-0791-9218

Gonzalez-Parra G

Gopal A

https://orcid.org/0000-0002-4683-5383

Gordon D

https://orcid.org/0000-0002-1090-9539

Gorzelak P

https://orcid.org/0000-0001-5706-1881

Goula M

Goutcher R

Goyret J

https://orcid.org/0000-0002-6509-4824

Gracia-Lázaro C

https://orcid.org/0000-0002-9769-8796

Graham K

https://orcid.org/0000-0002-7422-7676

Grandal D'Anglade A

Grant S

Gravena W

Greaves D

https://orcid.org/0000-0003-3906-9630

Green C

Griffin B

Griffin C

Griffin D

Griffith D

Gronfeldt Gunnarsson B

Groothuis T

https://orcid.org/0000-0003-0741-2174

Grossi PC

Grzybowski A

Gu L

Gu Y

Gudder S

Gudka M

https://orcid.org/0000-0001-6733-9381

Guess A

Guevara Fiore P

Guimarães N

Guin L

Gulizzi V

Gull N

Gundersen OE

https://orcid.org/0000-0002-9754-5941

Guo P

Gurrin L

Gustison M

Gutierrez L

Gutierrez-Ibanez C

https://orcid.org/0000-0002-0468-8223

Gutowsky S

Gårdmark A

https://orcid.org/0000-0003-1803-0622

Göritz A

Günther B

Haeffel G

https://orcid.org/0000-0002-4029-1493

Haghpanah F

https://orcid.org/0000-0002-2158-8486

Haifig I

https://orcid.org/0000-0003-4749-8945

Haig D

Haklay M

https://orcid.org/0000-0001-6117-3026

Halawa M

https://orcid.org/0000-0001-6358-2691

Hallermann J

Halliday W

Hamid S

Hammel J

https://orcid.org/0000-0002-6744-6811

Hammerschmidt K

Han F

https://orcid.org/0000-0003-3399-4008

Han J

Han Q

Handa N

Hanson B

https://orcid.org/0000-0002-1720-1656

Hao E

Haridy Y

Harper E

https://orcid.org/0000-0002-1092-3867

Harris D

Harussani MM

Harvey C

https://orcid.org/0000-0002-2830-0844

Hastings A

https://orcid.org/0000-0002-0717-8026

Hatton FL

Haucke M

https://orcid.org/0000-0002-8761-1124

He M

He W

https://orcid.org/0000-0001-9069-1447

He X

He Z

Heather F

Heck D

Heck T

Hellmann J

Hellmich C

Hensel L

Herberholz J

https://orcid.org/0000-0001-7584-5903

Hernandez A

https://orcid.org/0000-0003-3723-2828

Herrel A

Herrera Y

Herzog S

https://orcid.org/0000-0002-5373-5866

Hex S

Heyman T

https://orcid.org/0000-0003-0565-441X

Hilton C

Hines H

https://orcid.org/0000-0003-0299-5569

Hipfner M

Hisano G

Hoang H

Hobson E

https://orcid.org/0000-0003-1523-6967

Hoemann K

Hoffman J

Hofmann OT

Hogg C

https://orcid.org/0000-0002-6328-398X

Holland T

https://orcid.org/0000-0002-3537-5756

Hollanders M

https://orcid.org/0000-0003-0796-1018

Holleman G

Holtz T

Horinek D

Horowitz T

https://orcid.org/0000-0001-5571-6266

Horrocks N

https://orcid.org/0000-0003-0762-4142

Houdelier C

Houee G

Hove M

Howerton E

https://orcid.org/0000-0002-0639-3728

Hsu B-Y

https://orcid.org/0000-0002-3799-0509

Hu D

https://orcid.org/0000-0002-0017-7303

Hu Z

Huang C

Huang J

Huang X

https://orcid.org/0000-0001-9441-1473

Hudgins E

https://orcid.org/0000-0002-8402-5111

Huijben S

https://orcid.org/0000-0002-3537-1915

Huo L

Hurd P

Hutson J

Hyde J

Ichihara M

https://orcid.org/0000-0002-5591-7129

Ichinose G

https://orcid.org/0000-0002-3349-5869

Idriss S

Iftikhar H

Illán Castillo R

Im S

Imashiro C

https://orcid.org/0000-0002-0716-2585

Ingram PB

Innominato P

Ioannou C

https://orcid.org/0000-0002-9739-889X

Iori G

Iqbal M

Iqbal MW

Isager P

https://orcid.org/0000-0002-6922-3590

Ishihara J

Ishihara K

Ishii Y

Iswandaru D

Itsko M

Ivanovic A

Ivimey-Cook E

https://orcid.org/0000-0003-4910-0443

Ivory J

Jablonszky M

Jackson I

Jacquemet V

Jaeggi A

https://orcid.org/0000-0003-1695-0388

Jagiello Z

https://orcid.org/0000-0003-1606-2612

Jain V

Jambura P

James A

https://orcid.org/0000-0002-1543-7139

James K

https://orcid.org/0000-0003-3403-3268

Jana A

Janiak C

Janik V

https://orcid.org/0000-0001-7894-0121

Janjua MRSA

https://orcid.org/0000-0002-4323-1736

Janovský B

https://orcid.org/0000-0002-1375-2151

Janssens L

Janvier P

Jardine R

Jayapal R

https://orcid.org/0000-0003-3656-541X

Jenkins p

Jeong D

Jezek P

Jiang W

https://orcid.org/0000-0003-0953-5047

Jiang Z

Jiangzuo Q

https://orcid.org/0000-0003-4773-5349

Jiménez-Santos MA

https://orcid.org/0000-0002-1486-9351

Jiun YY

Johansen A

https://orcid.org/0000-0002-3531-7628

Johnston S

https://orcid.org/0000-0003-4532-5573

Jones DA

Jones N

https://orcid.org/0000-0002-6031-7507

Jordaan RK

Jordan J

Joshi A

Jowitt S

Joyce M

Jumabekov A

https://orcid.org/0000-0003-0051-9542

Kadokawa J

Kailasa SK

Kamber B

Kane A

https://orcid.org/0000-0002-2830-5338

Kanellopoulos A

Kang R

Kanngiesser P

https://orcid.org/0000-0003-1068-3725

Kao R

https://orcid.org/0000-0003-0919-6401

Kaondera-Shava R

https://orcid.org/0000-0002-4254-9777

Kapatsinski V

Karbasi S

Karimzadeh Soureshjani H

Karl J

Karsdorp F

https://orcid.org/0000-0002-5958-0551

Karyianni E

Kaul C

Kaullysing D

Kavak H

Kazemi M

Keglevich G

Kekecs Z

https://orcid.org/0000-0001-9247-9781

Kelley L

https://orcid.org/0000-0003-0700-1471

Kelly C

https://orcid.org/0000-0002-0693-7211

Kenrick D

Kenward B

Kersting D

Khalid H

Khan A

Khan Khalil AA

Khan MAM

Khan MJI

Khassetarash A

Khatib J

Khelifa R

https://orcid.org/0000-0001-6632-8787

Kibler A

Kim S

Kimmitt A

https://orcid.org/0000-0003-0044-8297

Kimock C

King G

https://orcid.org/0000-0002-5327-7631

Kingwell C

Kishi A

Kitao A

Kitchener A

https://orcid.org/0000-0003-2594-0827

Kittakoop P

Klages R

Klamser P

Klan F

https://orcid.org/0000-0002-1856-7334

Klapwijk E

https://orcid.org/0000-0002-8936-0365

Klapötke T

Kleshnina M

https://orcid.org/0000-0002-5518-8317

Kleynhans J

Klika V

https://orcid.org/0000-0001-6272-8396

Kmentova N

Knutsson P

Kohli M

Kohlmeier P

https://orcid.org/0000-0002-7123-9209

Koirala D

https://orcid.org/0000-0001-6424-3173

Kokol P

Kolomenskiy D

https://orcid.org/0000-0003-0107-6894

Kong D

Kortessis N

https://orcid.org/0000-0003-4690-7508

Korzhikova-Vlakh E

Koskela J

Kostić M

Kovalerchuk B

Kozbelt A

Kramp R

https://orcid.org/0000-0003-4832-2474

Krashennikova A

Krause A

https://orcid.org/0000-0001-9638-7278

Kreplins T

Kressler MM

https://orcid.org/0000-0003-3852-104X

Kristensen T

Kristjansson A

Kroliczak G

Krone M

Kronenberger J

Kronqvist J

Kross S

Kubo K

Kumar K

Kuntner M

Kupcu A

Kusakabe M

Kuzmin E

Kythreotis A

La Loggia O

https://orcid.org/0000-0003-4855-1269

LaBarbera K

https://orcid.org/0000-0002-2000-7280

Lachaud J-P

Lackey A

Lacquaniti F

Lallensack J

Lamur A

https://orcid.org/0000-0002-9977-0085

Lane S

https://orcid.org/0000-0002-3797-3178

Lang J

https://orcid.org/0000-0002-9530-2989

Lang M

https://orcid.org/0000-0002-2231-1059

Lange C

Lange Y

Largo A

Larson RG

LaRue M

Lautarescu A

Lavan N

Lavender S

Lawhorn K

Layek U

Layton K

Leardini A

Leavens D

Lebre PH

Lecorps B

https://orcid.org/0000-0001-5973-7152

Ledin C

Lee A

Lee CH

Lee E

https://orcid.org/0000-0003-2075-6342

Lee P

Lee Sang-H

Lee-Confer J

Leeb T

Leganes-Fonteneau M

Legare B

Legendre L

https://orcid.org/0000-0003-1343-8725

Lehnen L

Leinwand J

Leng X

https://orcid.org/0000-0002-0041-7602

Lenhard J

Lenninger S

Lesch R

https://orcid.org/0000-0001-7151-252X

Lettieri N

Leunissen J

Leverett E

https://orcid.org/0000-0001-6586-7359

Leverger K

Lewandowsky S

Lewis R

Li B

Li C

Li G

Li J

Li P

https://orcid.org/0000-0002-8299-9166

Liangzhi Z

https://orcid.org/0000-0001-7135-2378

Liebal K

https://orcid.org/0000-0003-2447-8327

Lieberman B

Liehrmann O

https://orcid.org/0000-0001-5390-8985

Lin D

https://orcid.org/0000-0003-4492-0944

Lin YC

Link A

https://orcid.org/0000-0003-3125-249X

Liu C

https://orcid.org/0000-0002-0381-4136

Liu J

Liu K

Liu M

Liu S

Liu Y

https://orcid.org/0000-0003-1112-1863

Livesey E

Llorente M

https://orcid.org/0000-0001-9003-1983

Lo LK

Locatello L

https://orcid.org/0000-0002-3530-2565

Lodeiro C

Lohan K

Longo Jr. L

Lopes AM

Lopes C

Lopez-Persem A

Loron C

https://orcid.org/0000-0002-3317-4029

Lovelace D

https://orcid.org/0000-0002-0154-4777

Lucchetti G

Luchiari A

https://orcid.org/0000-0003-3294-7859

Luna-Arias JP

Luong L

Luschi P

https://orcid.org/0000-0001-7081-3507

Luthfi OM

Lynch J

Lyon B

https://orcid.org/0000-0001-8733-9944

López-García JM

Lü L

https://orcid.org/0000-0001-7533-6244

M. Campos C

https://orcid.org/0000-0002-5151-6774

M. Silva LG

https://orcid.org/0000-0002-2329-5601

Mabrouki J

https://orcid.org/0000-0002-3841-7755

MacCarron P

MacLaren J

https://orcid.org/0000-0003-4177-227X

MacLean A

https://orcid.org/0000-0003-0689-7907

Macías García C

https://orcid.org/0000-0003-3242-4214

Madden J

https://orcid.org/0000-0002-0691-0967

Madigan D

Madsen A

Madzia D

Magioli M

https://orcid.org/0000-0003-0865-102X

Maglieri V

https://orcid.org/0000-0001-6631-043X

Mahmood T

Maialeh R

Maisey J

https://orcid.org/0000-0001-7566-0124

Majireck M

Makagon MM

Malard LA

Malcolm S

Malkowski P

https://orcid.org/0000-0003-0870-438X

Mallanna Nagaraja B

Mallick A

Mallott E

https://orcid.org/0000-0001-5446-8563

Malomo D

Mandal A

Mandeville C

https://orcid.org/0000-0002-1361-607X

Manica L

https://orcid.org/0000-0001-6005-7103

Mann J

Mansfield A

Margetts R

Marie A

Mariencheck C

Mariette M

https://orcid.org/0000-0003-0567-4111

Mariottini Y

Markelius A

Marks L

Marom A

https://orcid.org/0000-0003-0537-4661

Marques T

Marro M

https://orcid.org/0000-0002-6661-9637

Marshall BM

Martin A

https://orcid.org/0000-0002-2843-6928

Martin K

Martinelli A

https://orcid.org/0000-0003-4489-0888

Martinez G

https://orcid.org/0000-0003-2601-4876

Martínez-Olivas A

Marwayana O

Mashatola T

Maslov S

Matanza X

Mathanlal T

Mathews L

Matthews J

https://orcid.org/0000-0002-1895-3403

May Concha I

May K

https://orcid.org/0000-0001-8114-5989

May-Collado L

Mazzi V

Mazzone G

McCabe K

McDonough-Goldstein C

McGarvey D

McGowen M

McKiernan EC

https://orcid.org/0000-0002-9430-5221

McLennan EA

McManus C

https://orcid.org/0000-0003-3510-4814

McMenamin M

https://orcid.org/0000-0002-3764-0963

Meeinkuirt W

Meek PD

Mehler A

Mehta S

https://orcid.org/0000-0002-4889-5101

Mejia O

Meloro C

https://orcid.org/0000-0003-0175-1706

Menzel F

https://orcid.org/0000-0002-9673-3668

Mercier H

Merkel F

Metcalfe A

https://orcid.org/0000-0001-6101-7752

Meunier J

https://orcid.org/0000-0001-6893-2064

Meyer JFCA

Michael J

https://orcid.org/0000-0003-4516-196X

Michalczyk L

https://orcid.org/0000-0002-3174-0832

Michaud M

https://orcid.org/0000-0001-7075-9862

Mideo N

Mielke M

https://orcid.org/0000-0001-6328-0589

Millard M

Miller B

Miller C

https://orcid.org/0000-0002-3317-1821

Mishra A

Mishra SK

Mitchell E

https://orcid.org/0000-0001-6517-2231

Mittelstadt B

https://orcid.org/0000-0002-4709-6404

Miyashita T

Mizrahi M

Mizumoto N

https://orcid.org/0000-0002-6731-8684

Mkilima T

Mochizuki S

Mocho P

Mochurad L

https://orcid.org/0000-0002-4957-1512

Mohan CG

Moher D

https://orcid.org/0000-0003-2434-4206

Moiron M

Mollo DC

Momennia M

https://orcid.org/0000-0002-6184-240X

Mondal M

Monk J

https://orcid.org/0000-0002-5214-6576

Moor H

Moore P

Moorehouse K

Moots H

Moraes R

Moreno Fernández MM

Moreno MJ

Moreno-Rueda G

https://orcid.org/0000-0002-6726-7215

Mori K

Morlier J

Moser C

https://orcid.org/0000-0002-5068-0740

Moss R

https://orcid.org/0000-0002-4568-2012

Mouginot P

https://orcid.org/0000-0003-2967-065X

Mourot L

Mphahlele MJ

Mueldner G

Muletz C

Munday P

Muni SS

Murase Y

https://orcid.org/0000-0002-5872-420X

Murphy J

Murphy S

Murray M

https://orcid.org/0000-0002-2591-0794

Mushayabasa S

https://orcid.org/0000-0002-7423-2934

Muslim R

Mutono N

Muzaffar SB

Muñoz AG

Muñoz-Rojas J

Mwanga E

Müller R

https://orcid.org/0000-0002-4688-1515

N'Konou K

Nabawy M

https://orcid.org/0000-0002-4252-1635

Nagatsu M

Nagy M

Najem JS

Nakagawa Y

Nakata K

https://orcid.org/0000-0002-1207-3448

Nalla S

Nan Z

Narayan J

Nardi M

Naruphontjirakul P

https://orcid.org/0000-0001-9998-6700

Nasari H

Nasr JJ

https://orcid.org/0000-0002-2423-7315

Nastase S

Navarra J

Navazani S

Nawar AM

Nayak D

Nazir A

Ndaka A

Nebel C

https://orcid.org/0000-0002-0848-1676

Nehring V

https://orcid.org/0000-0002-0494-1428

Nelson F

Nelson L

Nemati-Anaraki L

Nersesov SG

Nestor A

user S

https://orcid.org/0000-0003-0305-1615

Neville F

Newton J

Nešporová K

Ngcamphalala C

Nguyen N

Nguyen T

Niarakis A

Nicholls S

Nie E

https://orcid.org/0000-0003-1453-4460

Nielsen H

Nieto C

Nneji LM

Nores C

https://orcid.org/0000-0002-3042-1960

Norgaard M

Nosarti C

Novotny V

https://orcid.org/0000-0001-7918-8023

Nussbaum C

O' Sullivan C

O'Connell N

O. Dull R

Oberst S

https://orcid.org/0000-0002-1388-2749

Obracaj D

Occelli V

https://orcid.org/0000-0003-3972-0526

Ocklenburg S

Odriozola I

Oestreich W

Ogden N

https://orcid.org/0000-0002-1062-7283

Oh E

Ohmer M

https://orcid.org/0000-0002-5937-6585

Okafor E

Okesola B

Oliveira G

https://orcid.org/0000-0002-7210-6408

Olmos LE

Ontiveros V

https://orcid.org/0000-0001-8477-2574

Opaev A

Oppel S

Orio A

https://orcid.org/0000-0002-5566-6139

Ortega-Castro J

Ortego J

Ortman S

Osiecka AN

https://orcid.org/0000-0001-5392-7895

Osthoff G

Oswald J

https://orcid.org/0000-0002-1524-9592

Oswald L

Otero A

https://orcid.org/0000-0002-4766-7086

Otero M

Ottewell K

Otti O

Oxner M

Oyewole GJ

https://orcid.org/0000-0001-7597-887X

O’Meara D

https://orcid.org/0000-0003-1372-5505

Paarporn K

Pacholski A

Paiva V

Pal R

Pale U

Palottini F

Panigrahy AK

Panzetta V

https://orcid.org/0000-0001-6174-4116

Papachatzis N

Papadopoulos K

https://orcid.org/0000-0003-1042-6016

Papudeshi B

Park H-J

Park K

Parks S

https://orcid.org/0000-0001-6663-627X

Parra Cordova A

https://orcid.org/0000-0003-3807-6230

Parry LA

Partanen M

Pascal R

Pasquali L

Passarotto A

https://orcid.org/0000-0002-6661-8714

Patel H

https://orcid.org/0000-0003-4740-7329

Patriarca M

Pau G

Paul G

https://orcid.org/0000-0002-4493-6092

Paul S

Paulun V

Pauly G

Pause B

https://orcid.org/0000-0003-0471-2550

Pawson S

Payne N

https://orcid.org/0000-0002-5274-471X

Peart R

Peckre L

Percino MJ

Pereira R

https://orcid.org/0000-0002-5105-5082

Perilloux C

Perlman M

https://orcid.org/0000-0002-1269-3882

Perrusquía A

Perry G

Peshkov I

Petrone N

Petropoulos G

Pezzotti S

Phani V

Philpot R

https://orcid.org/0000-0002-0359-2123

Philson C

https://orcid.org/0000-0002-5974-347X

Phitsuwan P

Piazza RD

Piccini G-M

Picin A

Pilastro A

https://orcid.org/0000-0001-9803-6308

Pillet H

Pineda M

Pinheiro D

Pinti J

https://orcid.org/0000-0002-0664-0936

Piot O

Piovia-Scott J

Pirotta E

https://orcid.org/0000-0003-3541-3676

Pittler M

Plötner M

Podos J

https://orcid.org/0000-0002-2546-7999

Poissenot K

https://orcid.org/0000-0003-4236-1421

Poleszczuk J

Poletto C

Pollack A

Polman E

Pombal M

Pons P

Popescu A

https://orcid.org/0000-0001-8122-4134

Posillico M

Post A

Post S

https://orcid.org/0000-0003-1091-9742

Pote L

Potticary A

https://orcid.org/0000-0002-1157-5315

Pottie IR

Powell D

Powell L

Prados F

Prainsack B

Prakash C

Prashanth GK

Prates L

Preiszner B

Prentiss A

https://orcid.org/0000-0002-9393-3170

Preston E

https://orcid.org/0000-0002-4098-2630

Price D

https://orcid.org/0000-0003-0076-3123

Prike T

https://orcid.org/0000-0001-7602-4947

Pringle WJ

Prokopenko C

Pross A

Pérez-Rosales G

https://orcid.org/0000-0001-6577-3416

Pérez-Staples D

Qian S

Quijada-Rodriguez A

R Mariselvam

Racicot R

Rahman I

https://orcid.org/0000-0001-6598-6534

Raia P

https://orcid.org/0000-0002-4593-8006

Rajagopal V

https://orcid.org/0000-0002-5509-402X

Rajarajan A

Rajendran V

Rakesh KP

Ramos J

Ramprasadh C

Ramírez Zora DE

Rana AM

Rana M

Rasambainarivo F

Ratsimbazafindranahaka M

https://orcid.org/0000-0001-8916-237X

Rault A

Ravosa M

Rayfield K

https://orcid.org/0009-0003-5702-6770

Razum J

Rebotim A

Recio Saucedo A

Reddy BV

Remili A

Rempel EL

Rengaswami S

Restif O

https://orcid.org/0000-0001-9158-853X

Rey-Villiers N

Reyne B

https://orcid.org/0000-0002-4899-1240

Reynisson J

Rezk MR

Riekkola L

Rizzuto M

https://orcid.org/0000-0003-3065-9140

Robert D

Roberto-Neto O

Robinson E

https://orcid.org/0000-0002-1299-6541

Robinson G

Robinson J

Robson S

Rodrigues C

Roeder K

https://orcid.org/0000-0002-2628-5003

Rohlfs M

https://orcid.org/0000-0002-8767-1629

Romashchenko A

Romero SE

Román Caballero R

Rong Z

Roozenbeek J

https://orcid.org/0000-0002-8150-9305

Roper J

Rosado AM

Rossi F

Rossignac-Milon M

Roth A

Roth T

https://orcid.org/0000-0002-2985-120X

Roth-Nebelsick A

Rougier M

Rowe M

Rowell M

Rowlands A

Rowley-Conwy P

Roy D

Ruan R

Rub MA

Rubalcaba J

https://orcid.org/0000-0003-4646-070X

Ruhnau P

https://orcid.org/0000-0001-6546-7312

Rule J

https://orcid.org/0000-0001-7256-2393

Ruoff P

Rusconi E

https://orcid.org/0000-0003-2700-205X

Ryan M

https://orcid.org/0000-0003-2373-4384

Ryder M

https://orcid.org/0000-0002-1363-8148

Römer H

https://orcid.org/0000-0003-4326-0470

Rönkä K

https://orcid.org/0000-0003-4450-676X

Röttger P

Sa B

Sadhu SD

Sadras V

https://orcid.org/0000-0002-5874-6775

Saggiomo V

Saha S

https://orcid.org/0000-0002-1207-2525

Sahin D

Saikia AK

Saint-Aubin J

Sainte-Marie M

Saiz H

Sakineh Omidi S

Sakura M

https://orcid.org/0000-0002-0857-7176

Saladino GM

https://orcid.org/0000-0002-6854-1423

Salahinejad A

Salavati-Niasari M

https://orcid.org/0000-0002-7356-7249

Samanta A

Sanchez Amaro A

Sandifer P

Sandkam B

https://orcid.org/0000-0002-5043-9295

Sannassy Pilly S

https://orcid.org/0000-0002-9802-7759

Santicchia F

https://orcid.org/0000-0003-4814-9632

Santos FP

https://orcid.org/0000-0002-2310-6444

Santos R

Sarafoglou A

Sarkar S

https://orcid.org/0000-0003-3990-6128

Sartori G

Sastry N

Sawerwain M

Sayigh L

https://orcid.org/0000-0001-8334-1326

Scerri M

https://orcid.org/0000-0001-9036-2619

Schellenberg G

https://orcid.org/0000-0003-3681-6020

Scheumann M

https://orcid.org/0000-0002-4091-9500

Scheun J

Schimit PHT

Schlupp I

https://orcid.org/0000-0002-2460-5667

Schmalz X

Schmid D

Schmidt BVKJ

Schmidt J

https://orcid.org/0000-0001-8549-0587

Schneider J

https://orcid.org/0000-0001-8523-7354

Schnier C

https://orcid.org/0000-0001-7238-7871

Schoenle A

Schrock JM

Schulte C

Schulting R

Schultner E

https://orcid.org/0000-0002-5069-9732

Schulz A

https://orcid.org/0000-0001-8007-5157

Schulze I

https://orcid.org/0000-0002-4539-2592

Schuwerk T

https://orcid.org/0000-0003-3720-7120

Schwaminger SP

Schwan A

https://orcid.org/0000-0003-3362-9633

Schwarz K

Schweinsberg M

Schweizer S

https://orcid.org/0000-0001-6153-8291

Schäfer M

Scott C

https://orcid.org/0000-0003-0860-4805

Scott R

Scott-Samuel N

https://orcid.org/0000-0002-8270-8437

Seale H

Secomb TW

Seki Y

https://orcid.org/0000-0003-2919-3048

Sellitto M

Senar JC

https://orcid.org/0000-0001-9955-3892

Sengupta D

Seo R

Sepp T

https://orcid.org/0000-0002-8677-7069

Seth SK

Seto J

Sewall K

https://orcid.org/0000-0002-9347-5878

Sexton C

https://orcid.org/0000-0003-0174-7459

Seyfried A

Sha J

Shabbir W

https://orcid.org/0000-0002-0753-7850

Shackell N

Shafik W

Shah DA

Shakoori AR

Shams TA

Shanker NR

Shanmugasundaram A

Shannon G

https://orcid.org/0000-0002-5039-4904

Sharief A

Sharma VK

Shawkey M

https://orcid.org/0000-0002-5131-8209

Shefelbine S

Sheng W

Shi J

Shi Z

https://orcid.org/0000-0003-2388-6695

Shibasaki S

Shigeki Matsunaga S

Shigeta Y

Shruthi J

Shu T

Shukla S

Shultz A

Sibaroni Y

Siciliano-Martina L

Siciliano-Martina Le

Siebert MJ

https://orcid.org/0000-0003-4385-5773

Signorelli F

Sila-Nowicka K

Silva-Parades G

Silvatti A

Simons G

https://orcid.org/0000-0002-9858-4088

Singh A

Singh S

https://orcid.org/0000-0003-3817-8819

Sinsch U

Siqueira F

Siracusa ER

https://orcid.org/0000-0003-4205-7278

Sireci M

Sirois F

Sit T

Sitzia S

Siu-Wing Chan A

Skibsted J

Smallegange I

Smeele S

https://orcid.org/0000-0003-1001-6615

Smit N

https://orcid.org/0000-0003-0440-1998

Smith E

Smith I

Smith J

https://orcid.org/0000-0002-3342-4454

Smith J

Smith M

Smith MD

Sneed J

Snigirova AO

Snover M

Sobhanardakani S

Sobral G

Socha J

https://orcid.org/0000-0002-4465-1097

Soldati A

Sommer-Trembo C

https://orcid.org/0000-0001-9952-2906

Son M

Song C

https://orcid.org/0000-0001-7490-8626

Sonmezoglu S

Soras R

Sorensen P

Soto C

Souza T

Soylak M

Spaiser V

Spedding G

http://orcid.org/0000-0003-3033-7897

Spence A

Sperone E

Spicer S

https://orcid.org/0000-0001-7585-8886

Spies I

Spinello D

Sprenger R

Sreedhara S

Sresakoolchai J

Srinivasan M

https://orcid.org/0000-0002-7811-3617

Srinivasan Rajsri K

https://orcid.org/0000-0003-2877-3542

Srivastav AK

Stanton J

Stark H

Starr R

Steenman M

https://orcid.org/0000-0003-4547-4448

Stefan C

Steiner U

https://orcid.org/0000-0002-1778-5989

Stevenson P

Stewart-Williams S

Stiver K

https://orcid.org/0000-0002-0650-8281

Stock S

https://orcid.org/0000-0002-8886-4035

Stoffregen T

Stone A

Struck T

Stulp G

https://orcid.org/0000-0003-0173-5554

Sturdy C

Su Z

Succar R

Suhendi E

Sujiono EH

Suliman FO

Sumida S

Sun B

https://orcid.org/0000-0002-1826-0770

Sun G

Sun L

https://orcid.org/0000-0002-8112-2055

Surapaneni V

https://orcid.org/0000-0002-6241-9048

Sutton J

Suzuki S

Svorligkou G

https://orcid.org/0000-0002-1472-0282

Swanson D

Sweeny A

https://orcid.org/0000-0003-4230-171X

Syed M

Szekely G

Szubielska M

Szulkin M

https://orcid.org/0000-0002-7355-5846

Szöllősi G

Sônego M

Tagu J

https://orcid.org/0000-0003-2331-7433

Tajik S

Takembo CN

Takemura A

https://orcid.org/0000-0002-5216-4969

Tamè L

Tang JW

Tang Q

Taniguchi I

Taniguchi S

Tanimoto J

Tanner J

https://orcid.org/0000-0002-7047-7256

Taurozzi A

Taylor J

Tello-Ramos M

https://orcid.org/0000-0003-0945-3498

Teng S

Ternon E

Teske A

Tettamanti G

https://orcid.org/0000-0002-0665-828X

Thanh ND

Thatcher H

Thawley C

Thelwell M

Thomases B

https://orcid.org/0000-0001-8502-8915

Thompson D

Thompson González N

https://orcid.org/0000-0002-3195-1277

Thorne L

Thornton I

Thorogood R

https://orcid.org/0000-0001-5010-2177

Thumsová B

Thuy B

https://orcid.org/0000-0001-8231-9565

Tierney A

Tilic E

https://orcid.org/0000-0003-0463-322X

Tizzoni M

https://orcid.org/0000-0001-7246-2341

Tjahjono DH

Todorov O

https://orcid.org/0000-0002-0295-7557

Tokumoto T

Tokuyama N

https://orcid.org/0000-0003-2566-1887

Tomkins B

Tonelli B

Torreta JP

Toscano M

https://orcid.org/0000-0001-8185-3002

Transtrum M

Trapote E

https://orcid.org/0000-0002-1165-3379

Travouillon K

https://orcid.org/0000-0003-1734-4742

Trawinski T

Trefzer M

Tricas T

Trindle C

Trujillo L

https://orcid.org/0000-0002-1299-0350

Tryjanowski P

https://orcid.org/0000-0002-8358-0797

Tsuchimatsu T

https://orcid.org/0000-0003-1241-9338

Tsurushima A

https://orcid.org/0000-0003-2711-297X

Tubiana L

Tuncgenc B

Tuni C

https://orcid.org/0000-0002-7190-1143

Türtscher J

Uchihashi T

Uchmański J

Ullah Mughal E

https://orcid.org/0000-0001-9463-9398

Unkelbach C

Upender G

Uszko W

Vad J

Valdes-Parada FJ

Valente D

https://orcid.org/0000-0001-6086-5135

van den Heuvel J

van der Geer A

van der Weiden A

van Loon A

van Oort H

Van Parijs S

Van Thanh D

https://orcid.org/0000-0002-8870-6934

Van Yperen J

Vancouver J

Vanoye L

Vargas A

Vargas-Garcia JR

Vassena N

https://orcid.org/0000-0001-5411-4976

Vasta M

Vaz PD

Vega A

Vega F

Velarde D

Veprauskas A

Vergne T

Verhagen H

Vermeij G

https://orcid.org/0000-0002-6450-5687

Vermolen F

https://orcid.org/0000-0003-2212-1711

Vernouillet A

https://orcid.org/0000-0003-2525-2936

Verster R

Vezzoli A

Viale R

Vidal D

https://orcid.org/0000-0002-6054-1357

Vidiella B

https://orcid.org/0000-0002-4819-7047

Vigotsky A

Viguerie A

Vilanova E

Villalobos-Segura E

Villastrigo A

Villemonteix T

Vilotijevic A

Vinn O

https://orcid.org/0000-0002-2889-2544

Visher E

https://orcid.org/0000-0003-3984-4748

Viswanathan H

Viteri M

Viñán-Ludeña MS

https://orcid.org/0000-0003-2692-5899

Vodeneev V

Vogeli B

https://orcid.org/0000-0003-1176-3137

Vohland K

von Cossel M

von Flach C

Vowles A

Voy B

Vranka M

Vreden C

Vriend S

Vulchanova M

Vázquez-Domínguez E

https://orcid.org/0000-0001-6131-2014

Wadie M

https://orcid.org/0000-0001-7467-7469

Wahyuningrum D

https://orcid.org/0000-0003-0638-1839

Wainwright DK

Wakeling James

Walker C

Walker N

Walker RL

Wallace R

Wallau G

Wallimann-Helmer I

Walsh M

Walsh S

Wang B

Wang J

Wang S

Wang X

https://orcid.org/0000-0001-6825-1031

Wang Z

Ward M

Watling D

Wauters L

https://orcid.org/0000-0002-4871-5035

Webb G

https://orcid.org/0000-0003-4210-0280

Weinrich M

Weissburg M

Weitzel D

Weitzel E

Wellman C

https://orcid.org/0000-0001-7511-0464

Wenzel T

Werner KM

West J

https://orcid.org/0000-0001-9579-4664

Westbury M

https://orcid.org/0000-0003-0478-3930

White P

https://orcid.org/0000-0002-9080-6678

Wilkin S

Will C

Willems E

William M

Williams H

https://orcid.org/0000-0003-0947-6243

Williams M

Williams S

https://orcid.org/0000-0001-7860-8962

Willwacher S

Wilmot M

Wingen T

https://orcid.org/0000-0002-1559-859X

Winkelmann S

Winsberg E

Winters S

Wittig B

https://orcid.org/0000-0003-1398-7425

Wittmann M

Wolf L

Wolfgang Schubert T

Wong A

https://orcid.org/0000-0001-9360-429X

Wong CM

Wood R

https://orcid.org/0000-0002-0165-5987

Woodrow C

Woolley T

https://orcid.org/0000-0001-6225-5365

Worsley S

https://orcid.org/0000-0003-4736-0938

Wouters P

https://orcid.org/0000-0002-4324-5732

Wu C

Wu D

Wu J

https://orcid.org/0000-0003-3509-5110

Wu X

Wu Y

Wu Z

Wystrach A

Xia C

https://orcid.org/0000-0003-2686-5072

Xiao B

Xiao Y

Xie Y

Xu D

Xu G

Yagi K

Yamaguchi T

https://orcid.org/0000-0002-5063-9924

Yamamoto K

Yamana T

https://orcid.org/0000-0001-8349-3151

Yang H

https://orcid.org/0000-0001-8778-1695

Yang H

Yang Y

Yao C-F

Yao Y

Yavari A

https://orcid.org/0000-0002-7088-7984

Yavuz M

https://orcid.org/0000-0003-3452-8551

Yazdi M

Yin Z

https://orcid.org/0000-0002-9391-0446

Yobom O

Yoccoz N

Yoo H

Yoo SB

Yorzinski J

https://orcid.org/0000-0002-4193-6695

Young B

Yu H

https://orcid.org/0000-0003-4462-4010

Yu L

Yun H

https://orcid.org/0000-0003-4211-3452

Yáñez F

https://orcid.org/0000-0002-9598-0616

Zaal F

Zachreson C

https://orcid.org/0000-0002-0578-4049

Zaher H

https://orcid.org/0000-0002-6994-489X

Zahid AN

https://orcid.org/0000-0001-6881-9766

Zambo D

Zanchi S

Zanella M

https://orcid.org/0000-0001-8456-5866

Zarrouk A

Zelepukin IV

Zeman S

Zendehboudi S

Zeng L-Q

Zerbini A

https://orcid.org/0000-0002-9776-6605

Zhang C

Zhang H

Zhang S

https://orcid.org/0000-0001-9900-5570

Zhang T

Zhang F

https://orcid.org/0000-0002-3643-018X

Zhao G

Zhao J

Zhao L

Zhao X

Zheng Z

Zhou J-W

Zhu L

Zhuravlev A

https://orcid.org/0000-0003-1611-6916

Zinchenko A

Zipple MN

https://orcid.org/0000-0003-3451-2103

Zou J-J

Zwijnenburg M

Zych M

https://orcid.org/0000-0001-6374-9498

